# Meta-Analysis and Multivariate GWAS Analyses in 80,950 Individuals of African Ancestry Identify Novel Variants Associated with Blood Pressure Traits

**DOI:** 10.3390/ijms24032164

**Published:** 2023-01-21

**Authors:** Brenda Udosen, Opeyemi Soremekun, Abram Kamiza, Tafadzwa Machipisa, Cisse Cheickna, Olaposi Omotuyi, Mahmoud Soliman, Mamadou Wélé, Oyekanmi Nashiru, Tinashe Chikowore, Segun Fatumo

**Affiliations:** 1 The African Computational Genomics (TACG) Research Group, MRC/UVRI and LSHTM, Entebbe 7545, Uganda; brendaumoh6@gmail.com (B.U.); soremekun.opeyemi@mrcuganda.org (O.S.); abramkamiza@gmail.com (A.K.); 2 The African Center of Excellence in Bioinformatics of Bamako (ACE-B), University of Sciences, Techniques and Technologies of Bamako, Bamako 3206, Mali; cheickna2@yahoo.fr (C.C.); mamadou.wele@fulbrightmail.org (M.W.); 3 H3Africa Bioinformatics Network (H3ABioNet) Node, Centre for Genomics Research and Innovation, NABDA/FMST, Abuja 901101, Nigeria; oyekan.nash@gmail.com; 4 Molecular Bio-Computation and Drug Design Laboratory, School of Health Sciences, Westville Campus, University of KwaZulu-Natal, Durban 4001, South Africa; soliman@ukzn.ac.za; 5 Malawi Epidemiology and Intervention Research Unit, Lilongwe P.O. Box 46, Malawi; 6 Hatter Institute for Cardiovascular Diseases Research in Africa (HICRA), Department of Medicine, University of Cape Town, Cape Town 7701, South Africa; taffymach@gmail.com; 7 Population Health Research Institute, David Braley Cardiac, Vascular and Stroke Research Institute, Hamilton, ON L8L 2X2, Canada; 8 Department of Biological Sciences, Faculty of Sciences and Techniques, University of Sciences, Techniques and Technologies of Bamako, Bamako 3206, Mali; 9 Institute for Drug Research and Development, S.E. Bogoro Center, Afe Babalola University, Ado Ekiti 360101, Nigeria; olaposi.omotuyi@abuad.edu.ng; 10 Sydney Brenner Institute for Molecular Bioscience, Faculty of Health Sciences, University of the Witwatersrand, Johannesburg 2050, South Africa; tinashe.chikowore1@wits.ac.za; 11 MRC/Wits Developmental Pathways for Health Research Unit, Department of Pediatrics, Faculty of Health Sciences, University of the Witwatersrand, Johannesburg 2050, South Africa; 12 Segun Fatumo, Department of Non-Communicable Disease Epidemiology, London School of Hygiene and Tropical Medicine, London WC1E 7HT, UK

**Keywords:** systolic blood pressure, diastolic blood pressure, GWAS, high blood pressure, multivariate, univariate

## Abstract

High blood pressure (HBP) has been implicated as a major risk factor for cardiovascular diseases in several populations, including individuals of African ancestry. Despite the elevated burden of HBP-induced cardiovascular diseases in Africa and other populations of African descent, limited genetic studies have been carried out to explore the genetic mechanism driving this phenomenon. We performed genome-wide association univariate and multivariate analyses of both systolic (SBP) and diastolic blood pressure (DBP) traits in 80,950 individuals of African ancestry. We used summary statistics data from six independent cohorts, including the African Partnership for Chronic Disease Research (APCDR), the UK Biobank, and the Million Veteran Program (MVP). FUMA was used to annotate, prioritize, visualize, and interpret our findings to gain a better understanding of the molecular mechanism(s) underlying the genetics of BP traits. Finally, we undertook a Bayesian fine-mapping analysis to identify potential causal variants. Our meta-analysis identified 10 independent variants associated with SBP and 9 with DBP traits. Whilst our multivariate GWAS method identified 21 independent signals, 18 of these SNPs have been previously identified. SBP was linked to gene sets involved in biological processes such as synapse assembly and cell–cell adhesion via plasma membrane adhesion. Of the 19 independent SNPs identified in the BP meta-analysis, only 11 variants had posterior probability (PP) of > 50%, including one novel variant: rs562545 (*MOBP*, PP = 77%). To facilitate further research and fine-mapping of high-risk loci/variants in highly susceptible groups for cardiovascular disease and other related traits, large-scale genomic datasets are needed. Our findings highlight the importance of including ancestrally diverse populations in large GWASs and the need for diversity in genetic research.

## 1. Introduction

Blood pressure (BP) is a quantitative trait that is affected by multifactorial genetic and environmental factors [1,2,3]. The heritability of high blood pressure is estimated to be 30–50% [4]. Elevated blood pressure, otherwise called hypertension, is the leading risk factor for many cardiovascular diseases such as stroke and coronary artery disease [5,6]. The global prevalence of hypertension among adults aged 30–79 years increased significantly from 650 million in 1990 to 1.28 billion in 2019, with two-thirds of this burden coming from low- and middle-income countries (LMICs) [7]. When compared to other ethnic groups, African Americans and others of African ancestry show a higher occurrence of high blood pressure [8,9,10,11].

Despite the global rise in the disease burden among individuals of African ancestry, limited genome-wide association studies (GWASs) of blood pressure traits have been conducted or included individuals of African ancestry [12,13,14]. For instance, the largest GWAS of blood pressure conducted to date in approximately a million individuals was predominantly composed of Europeans [15]. Additionally, only ~62% of all the genome-wide significant loci from this GWAS had the concordant direction of effects for individuals of African ancestry and moderate Pearson correlation coefficients with effect estimates in Europeans r2 = 0.37 in Africans, compared to the strong r2 = 0.78 for South Asians [15,16,17,18]. Another example is that the majority of blood pressure GWASs conducted in African-ancestry populations have small sample sizes [19,20,21,22,23] and they mostly use a single-trait approach without giving due consideration to phenotypic relatedness and the relationship between the two traits (SBP and DBP), which is a possible link between risk-related clinical measures and arterial properties [24,25]. Thus, many novel insights into blood pressure traits in people of African ancestry remain to be discovered.

Furthermore, various GWAS reports have shown that the genetic determinants of blood pressure have small effect sizes and vary significantly between European and non-European populations [26]. Therefore, our study aimed to extensively study the African population to better understand the genetic epidemiology underlying blood pressure traits in individuals of African descent. We also performed a multivariate GWAS in the hope that it would increase our study’s statistical power over the univariate approach and consequently increase the overall number of novel loci observed in our study.

**Figure 1:**
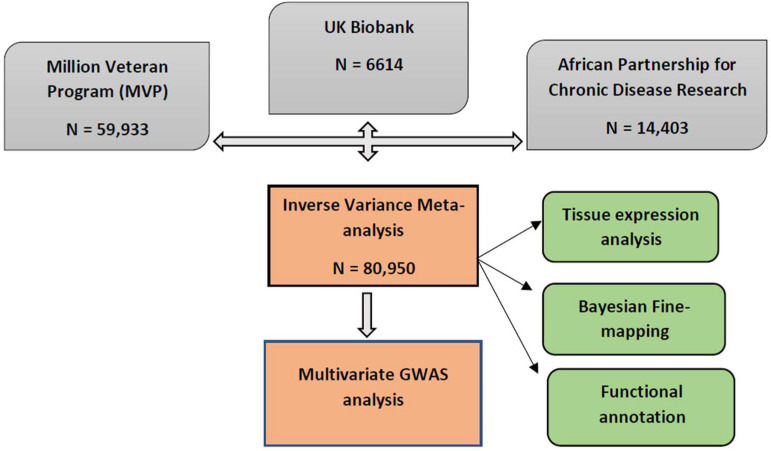
Study design schematic for discovery and validation of loci. APCDR; African Partnership for Control of Disease Research, UKB; United Kingdom Biobank, MVP; Million Veteran Program.

We conducted the largest GWAS of blood pressure in over 80,950 people, drawn from the African Partnership for Chronic Disease and Research (APCDR), African-ancestry individuals from the United Kingdom (UK Biobank), and the Million Veteran Program (MVP) in this study. Figure 1 depicts the overall study design; we used fixed-effects meta-analysis across the cohorts. We then performed a multivariate analysis, fine-mapping, and pathway and tissue enrichment test analysis to highlight relevant biological processes and investigate causal relationships with disease traits.

## 2. Results

### 2.1. Results Overview

We compiled GWAS summary statistics from three cohorts (Table 1), totaling 80,950 people of African descent. We used a univariate meta-analysis and a multivariate GWAS to find genetic variants linked to BP traits. At a genome-wide significant threshold of (*p* < 5 × 10^−8^) for both known and novel loci; the meta-analysis and multivariate approaches identified both known and novel loci. We used FUMA and fine mapping to gain more insight into the likely causal variants and molecular mechanism(s) that contribute to the genetics of BP traits.

### 2.2. Univariate GWAS Meta-Analysis

**Figure 2:**
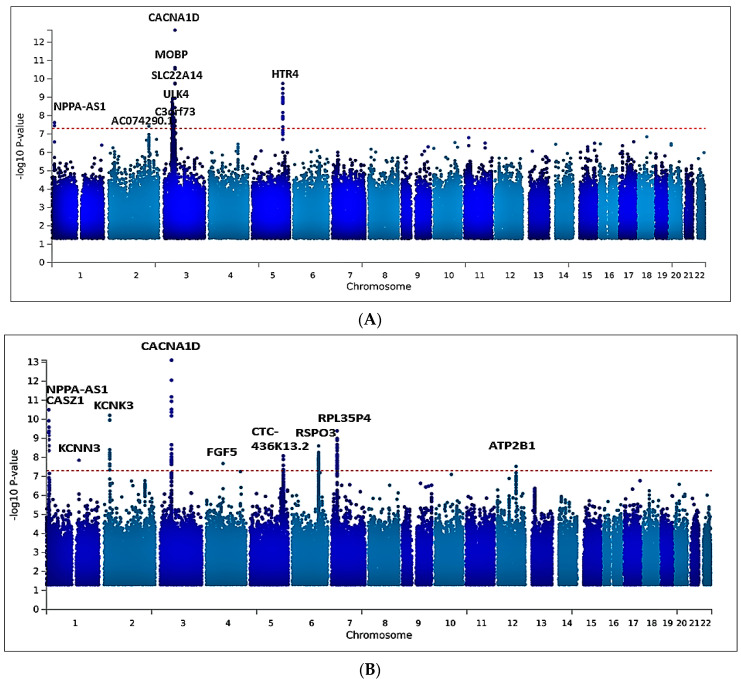
Manhattan plots showing the minimum *p*-value for the association across (**A**) DBP and (**B**) SBP blood pressure traits, computed using inverse-variance fixed-effect meta-analysis from 75,850 individuals. Each point on the Manhattan plots denotes a variant, with the *X*-axis representing the genomic position and the *Y*-axis representing the association level −log 10 (*p*-value). The horizontal red line shows the genome-wide significance threshold *p*-value = 5 × 10^–8^.

Meta-analysis of all six cohorts (n = 80,950) identified 166 significant variants for SBP (Supplementary Table S1) and 184 genome-wide significance variants (*p* < 5 × 10^−8^) for DBP (Supplementary Table S2). The significant SNPs for both blood pressure traits were clumped at ±500 Kb distance, leaving 10 for SBP (Supplementary Table S3) and 9 lead SNPs for DBP (Supplementary Table S4). After clumping, out of the 19 SNPs identified across both traits, 2 were at least 1 Mbp away from any previously reported BP locus and therefore considered novel: an intergenic variant rs77534700 in *AC074290.1* (*p* = 3.749 x 10^−8^) and rs562545, an intronic variant at *MOBP* (*p* = 1.823 x 10^−9^); both are associated with the DBP trait (Table 2, Figure 2A,B). Commonly known variants in *CACNA1D, HTR4, SLC22A14, NPPA-AS1, C3orf73, KCNK3, RPL35P4, CASZ1, NPPA-AS1, CTC-436K13.2, KCNN3, RSPO3, ATP2B1, FGF5, ULK4,* and *NPPA-AS1* were associated with SBP and DBP (Supplementary Tables S3 and S4).

The *CACNA1D* gene is an intron variant that has previously been identified in other populations, including African Americans, and is thought to regulate the renin–aldosterone–angiotensin system. The previously observed associations of the genetic variant in the meta-analyses were predominantly from the MVP cohorts, which might be driven by the fact that the largest proportion of our sample size came from the MVP’s African American population. We plotted the resulting *p*-values from this association analysis on a Manhattan plot. (Figure 2).

### 2.3. Functional Mapping and Annotation Analyses from FUMA of the Meta-Analysis

Using the default parameters on FUMA, we performed functional annotation on all SNPs in linkage disequilibrium (LD) to annotate and prioritize genes obtained from our meta-analysis. FUMA’s SNP2GENE function revealed that the majority of the markers in SBP were intergenic, followed by those in the intronic region (Supplementary Table S5). In DBP, the most significant portion of the markers were intronic SNPs, followed by intergenic SNPs (Supplementary Figure S1). We identified 19 genes through positional and/or eQTL mapping in SBP (Supplementary Table S6).

The MAGMA gene set, tissue expression, and pathway analyses were carried out as part of the FUMA workflow. According to the MAGMA gene set study, after Bonferroni correction, no DBP gene sets were significant, but 10 SBP gene sets were. SBP was specifically linked to gene sets involved in biological processes such as synapse assembly (including presynaptic membrane assembly and organization, postsynaptic density, and specialization assembly), cell–cell adhesion via plasma membrane adhesion molecules (i.e., the connection of one cell to another cell through the use of adhesion molecules that are at least partially embedded in the plasma membrane), and the Takada gastric cancer copy number (i.e., candidate genes in the regions of copy number loss in gastric cancer cell lines).

**Figure 3:**
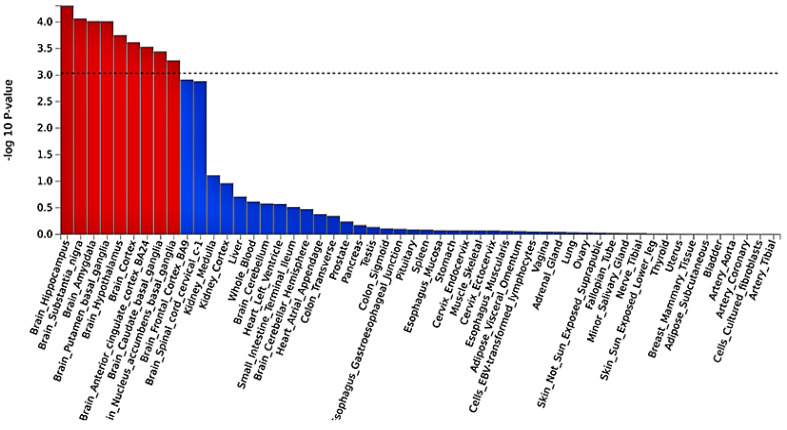
MAGMA tissue expression analysis using gene expression per tissue based on GTEx RNAseq data for 53 specific tissue types. Significant tissue is shown in red.

Based on MAGMA tissue expression analysis, SBP was not associated with any gene property analysis for tissue specificity. However, DBP was associated with nine tissue specificities significantly associated with brain tissues, particularly the hippocampus, brain substantia nigra, brain amygdala, brain putamen basal ganglia, hypothalamus, cortex, anterior cingulate cortex BA24, caudate basal ganglia, and nucleus accumbens basal ganglia (Figure 3). Notably, the strongest enrichment was observed for genes expressed in the hippocampus, followed by the putamen basal ganglia.

As part of the FUMA pipeline, we used GENE2FUNC to test differentially expressed genes (DEGs); DBP found no association with our GTEx v8 54 tissue types, but SBP found two significantly upregulated DEGs in the sigmoid and transverse colon (Supplementary Figure S2). Finally, we tested the enrichment of input gene sets (adjusted *p* < 0.05) and we found several gene sets previously associated with SBP, DBP, and correlated traits (Supplementary Figures S2–S8).

### 2.4. Fine-Mapping of Putatively Causal Variants

**Figure 4:**
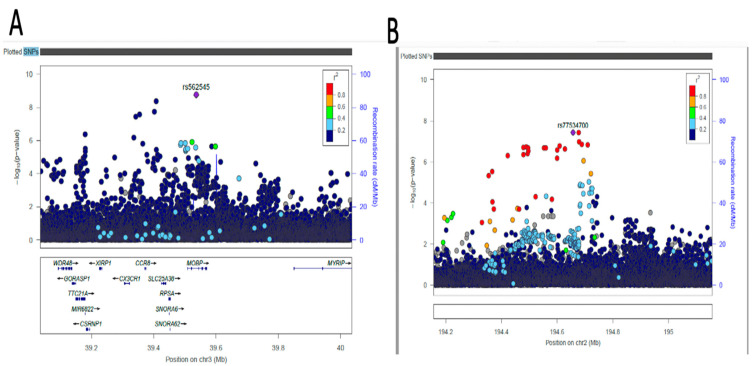
Regional visualization of the GWAS of −log10 of the *p*-value of genomic location MOBP (rs562545 in purple) (**A**) and AC074290.1 (rs77534700 in purple) (**B**), with each dot representing SNP on the corresponding genes at the bottom.

We performed Bayesian fine-mapping to pinpoint putative causal variants for distinct BP association signals using differences in the structure of LD between ancestry groups. Bayesian fine-mapping of the 19 distinct signals from the meta-analysis after clumping for DBP and SBP was undertaken in the region mapping 500 kb up- and downstream, which together accounted for 99% posterior probability and was based on association summary statistics from the meta-analysis GWAS. Only 11 variants from the lead SNPs had >50% posterior probability (PP) of being causal, including the novel variant rs562545 (MOBP, PP = 77%) (Supplementary Table S7, Figure 4) and known variants rs3821845 (*CACNA1D,* PP = 99%), rs12509595 (*FGFR,* PP = 99%), rs11129785 (*SLC22A14*, PP = 75%), rs12476527 (*KCNK3*, PP = 52%), rs5068 (*NPPA-AS1*, PP = 64%), rs73437338 (*ATP2B1*, PP = 52%), rs7720317 (*CTC-436K13.2*, PP = 59%), and rs1984285 (KCNN3, PP = 99%). One of the lead SNPs, rs880315 (*CASZ1*, PP = 95%), was not the lead variant in the fine-mapping but was overlapped by other variant rs17035646 (PP = 99%) (Supplementary Table S7).

### 2.5. Multivariate GWAS Analysis of Blood Pressure Traits Identifies Additional Novel Loci

**Figure 5:**
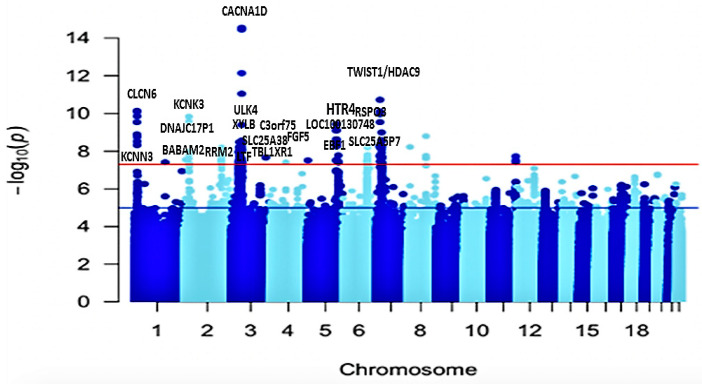
Manhattan plot showing *p*-values for the association computed from CPASSOC output results. Each point on the Manhattan plot denotes a variant, with the X-axis representing the genomic position and the *Y*-axis representing the association level −log 10 (*p*-value). The horizontal red line shows the genome-wide significance threshold *p*-value = 5 × 10^–8^.

Using CPASSOC, we performed a multivariate analysis. This method identified 166 genome-wide significant loci associated with blood pressure (Supplementary Table S8) (*p* < 5 × 10^−8^). After clumping, we identified 21 independent significant SNPs, 3 novel SNPs, and 18 known SNPs (Supplementary Table S9). Interestingly, using the model assuming heterogeneity in CPASSOC, we identified 3 novel independent significant variants (Table 3): rs138493856 (*DNAJC17P1/GLULP6*, *p* = 6.132 × 10^−09^), rs139235642 (*RRM2*, *p* = 2.798 × 10^−08^), and rs72619992 (*LOC105377644*, *p* = 1.134 × 10^−08^). The resulting *p*-values were then plotted and visualized in a Manhattan plot (Figure 5).

## 3. Discussion

This study describes the largest GWAS of blood pressure in African ancestry to date, involving a total of 80,950 individuals from the MVP, APCDR, and UK Biobank cohorts. The results of this analysis provide additional relevant information on the genetic and biological architecture of blood-pressure traits in people of African ancestry.

**Figure 6:**
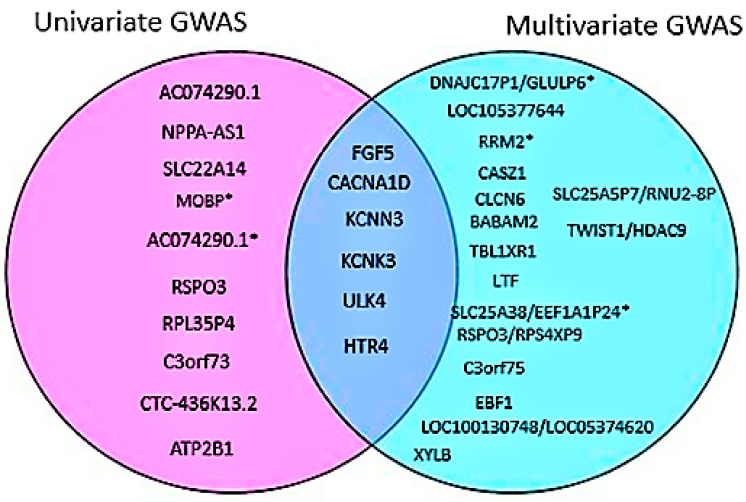
Venn diagram comparing the loci found by meta-analysis and multivariate GWAS analysis. Both methods identified known loci; the novel loci are indicated with an asterisk.

According to our results, the multivariate GWAS approach had greater statistical power in identifying new variants than the univariate meta-analysis (Figure 6). Previous GWAS studies had shown the power of the multivariate approach, especially when dealing with traits that are highly correlated [27].

Five novel variants were discovered using both methods. The multivariate approach identified three variants: *DNAJC17P1/GLULP6* (rs138493856), *RRM2* (rs139235642), and *LOC105377644* (rs72619992), while the univariate approach identified two variants: *AC074290.1* (rs77534700) and *MOBP* (rs562545). The *DNAJC17P1/GLULP6* gene, which is located in the intergenic region, is known to be associated with susceptibility to infectious disease [28] as well as educational attainment [29]. *RRM2* is a protein-coding gene that encodes one of two non-identical subunits for ribonucleotide reductase and is highly expressed in the bone marrow (28.1) and lymph nodes (20.5), along with other tissues [30]. High expression of this gene can lead to abnormal proliferation of histiocytes and can also be used as a marker for malignant changes in ovarian endometriosis [31]. The rs72619992 variant in *LOC105377644* is an uncharacterized RNA gene that belongs to the ncRNA class and does not code for any protein. In *AC074290.1*, our univariate method identified an uncharacterized pseudogene. According to the GWAS catalog, the *MOBP* gene, which is a myelin-associated oligodendrocyte-associated protein, is linked to Alzheimer’s disease [32], cognitive performance, and other brain-related disorders [33]. The *MOBP* gene is thought to be involved in both frontotemporal dementia and nervous system development. We used the largest BP summary statistics from European-ancestry individuals to look up our lead SNPs, while some of the lead SNPs were found to be replicated at *p*-value 0.05. None of the SNPs identified as being novel were replicated (Supplementary Table S10).

In the meta-analysis results, our in silico functional mapping and annotation analyses from FUMA revealed several biologically relevant signals. SBP gene sets, for example, were significantly associated with related biological systems, such as several synapse assembly components (including components correlated to nervous system development/neurons and chemical or electrical synapses), candidate genes in regions of copy number loss in gastric cancer cell lines, cell–cell adhesion via plasma membrane adhesion molecules (possibly part of action potentials generated by the movement of ions through transmembranous channels).

In addition, the SBP meta-analysis tissue enrichment analysis was associated with significantly upregulated DEGs in the sigmoid and transverse colon (Supplementary Figure S1), which may suggest that gut microbiota may play a role in the regulation of the gastro-renal axis and blood pressure [34]. Furthermore, the most interesting enrichment of input genes in gene sets significant in the Reactome was in the cardiac conduction and muscle contraction pathways for the SBP meta-analysis, which are the mechanisms and pathways that elicit rapid changes in heart rate and blood pressure and respond to changes in autonomic tone. On the other hand, our MAGMA DBP tissue expression analysis highlighted nine brain tissue types associated with DBP. For instance, the putamen, caudate, and nucleus accumbens basal ganglia are input nuclei as well as part of the corpus striatum, and the substantia nigra is a basal ganglia function-related nucleus; they are all involved in processing movement-related information. Dysfunction in this region is known to be associated with movement disorders like Huntington’s disease, as correlated by the GWAS catalog genes highlighted. In addition, the GWAS catalog genes included in the gene sets included blood pressure traits and their interactions with alcohol and cigarette smoking, hence these may be interesting environmental risk factors that should be investigated for their impact on BP traits in populations of African descent. Further investigation is needed to understand this, as different regions have different drinking and smoking habits.

Furthermore, our tissue expression analysis shows that DBP gene expression is enriched in the hippocampus (Figure 3), a brain region that is essential for learning and memory [35]. According to one study, hypertension is linked to decreased functional hippocampus connectivity and impaired memory [36]. As a result, more research is needed to understand our findings from in silico functional mapping and annotation analyses, as well as their mechanisms.

Our current study has several strengths. First, our study is the largest SBP and DBP GWAS meta-analysis of an African population; thus, it has allowed us to find novel loci and replicate prior findings. Secondly, our functional mapping and annotation found several biologically relevant regions that support our genetic findings, and these regions, tissues, and pathways are good candidates to explore further to elucidate the pathogenesis of blood pressure-related disorders like hypertension and prevent or treat them better. Finally, fine-mapping recommended target candidate loci to test in vivo and in vitro to improve our understanding of the regulators and genetic factors that affect blood pressure traits. The CPASSOC used for our multivariate GWAS increased statistical power and reflected the nature of the multivariate effect of traits on the genetic factor.

One of our limitations is that the “black” participants in our study are primarily from admixed regions with a variety of characteristics. Our study used a small sample size from the continental African population and this may be the reason why most of our variants were identified from the MVP dataset, as this data had the largest sample size (Table 3). Although our study is the largest study of SBP and DBP genetics, the overall sample size was small compared to contemporary GWASs for other traits. Thus, future studies will need to include more continental Africans to make sure our genetic risk factors can be used to make genetic risk scores that are inclusive of all or most African populations and their full range of diversity. Due to the diversity in African genomes, latent sub-structuring could inflate the results, but this effect was minimized by adjusting for principal components of the contributing cohorts in the GWAS model. Second, the paucity of functional genomics information specific to African people makes it challenging to evaluate the functional relevance of the relationships found. Thirdly, regional environmental factors, including dietary variations, variances in the prevalence of TB and HIV, and other non-communicable disease factors could potentially have an impact on BP outcomes; however, there is not enough research on these aspects in our target group. Afrocentric GWAS data are grossly limited, hence we used blood pressure GWAS data from individuals of African ancestry available and accessible to the authors.

In conclusion, we have conducted the largest GWAS of blood pressure in African ancestry, which has significantly enabled an in-depth understanding of its genetic component. Our analysis emphasizes the relevance of applying fine-mapping and multivariate methods to correlated traits and their increase in statistical power toward the discovery of causal variants. These strategies offer a reliable approach to better understanding the genetic epidemiology of blood pressure disease in individuals of African ancestry and treatment development strategy. Lastly, to better understand the implication of these results, future studies could replicate the results for the European population.

## 4. Materials and Methods

### 4.1. Study Population

The full description of the study population can be found in the Supplementary S1 cohort description, while the study design can be found in Figure 1.

### 4.2. Meta-Analysis of BP Summary Statistics in African-Ancestry Individuals

We aggregated BP association summary statistics across the three cohorts (UK Biobank, APCDR-UGR, DSS, DCC, and AADM, and the MVP) and performed an inverse-variance-weighted meta-analysis implemented in GWAMA [37]. We used a total of eighty thousand nine hundred fifty (80,950) sample sizes across the studied cohorts (Table 3). The resulting output was used for subsequent downstream analyses, and we then plotted the resulting *p*-value in a Manhattan plot.

### 4.3. Tissue Expression Enrichment Pathway Analysis

We performed a gene-based analysis with MAGMA 1.6 software (Multi-marker Analysis of Genomic Annotation) [38], which is available in FUMA [39]. MAGMA gene-based analysis is useful for analyzing and detecting multiple genetic markers in individuals with a weak effect, which is common in polygenic traits. The 1000 Genomes dataset was used as a reference to account for LD between SNPs, and the confounding effects of gene density and gene size were used as covariates. The pathway and tissue expression analyses were performed using the default parameters in FUMA, using the results obtained from the meta-analysis.

### 4.4. Functional Mapping and Annotation Analysis

We used an online functional mapping and annotation tool (FUMA) [39] to annotate SNPs from the GWAMA meta-analysis with their biological functionality, and then mapped them to genes using positional mapping and QTL association (blood eQTL) [40]. The independent SNPs were classified based on their *p*-values as genome-wide significant (*p* ≤ 5.0 × 10^−8^), their independence from each other (*r*^2^ < 0.1), and LD threshold within a 1 Mb window. Furthermore, the independent SNPs were annotated for functional effects on gene function using ANNOVAR [41]. For positional mapping, genes were mapped to SNPs if the physical distance between them was < 10 kb. The eQTL mapping used data from the blood cis-eQTL, and SNPs were mapped to genes on the premise that the SNPs had a significant effect on the expression of the gene. In addition, SNPs were filtered using a CADD score > 12.37, which is the threshold for deleterious scores (CADD scores are deleterious scores of genetic variants obtained by 63 functional annotations) [42]. Normalized gene expressions for 53 tissue types were obtained from GTEx. Other clumping parameters used were a reference panel to compute LD and MAFs (minor allele frequencies) = 1000 Genome project was used (AFR) [43]; minor allele frequency filter > 0.01; maximum distance between LD blocks to merge into a single locus for genomic risk loci = 250 kb; lead SNPs were classified as SNPs that were in LD with each other at r2 < 0.1.

### 4.5. Locus Definition

Lead SNPs from both univariate and multivariate analyses were defined based on positional mapping using 1 Mb; SNPs that had reached the genome-wide significant threshold (*p* < 5 × 10^−8^) were considered to be associated with BP. Loci were defined by flanking distance mapping 500 kb up- and downstream of peak SNPs, and we retained SNPs with the lowest *p*-value from both the meta-analysis and multi-trait analysis.

### 4.6. Fine-Mapping Analysis of Sentinel Variants

Following our output results from the multi-trait and meta-analyses, we performed Bayesian fine-mapping to identify possible causal variants for the locus ± 500 kb of all the lead SNPs. We used a Bayesian approach [44] to fine-map the loci of the lead SNPs. The Z-scores for the SNPs were then used to compute the Bayes factor for each SNP denoted as $BF_{i}$ , given by

$$BF_{i}=e^{\left[\frac{Z*Z-\log\left(K\right)}{2}\right]}$$

where *K* is the number of studies. The posterior probability of driving the association for each SNP was computed by

$$Posterior\;probability=\;\;\frac{BF_{i}}{\sum_{j}BF_{j}}$$

where the summation in the denominator is over all SNPs at the locus.

Ninety-nine percent credible set sizes were calculated by sorting all SNPs at the locus according to their posterior probability from highest to lowest and then counting the number of SNPs required to achieve a cumulative posterior probability greater or equal to 0.99. High confidence was defined as index SNPs that account for more than 50% of the posterior probability of driving the BP association at a given signal.

### 4.7. Multivariate GWAS Analysis

To further increase statistical power for discovery, we employed a cross-phenotype approach, implemented in CPASSOC software [45]. The cross-phenotype association analysis accounts for the correlation of summary statistics data among traits and the participating cohorts and allows for both heterogeneity and homogeneity effects. CPASSOC analysis generates two statistical tests: SHom and Shet, the latter of which is an extension of the former and improves statistical power when there is a difference in genetic effect sizes across traits. Meanwhile, the SHom test, which is similar to the fixed-effect meta-analysis approach, increases in power when the genetic effect sizes across the traits are the same.

## Figures and Tables

**Table 1:** Description of cohorts used in this study.

Cohort	Continent	Country	Sample Size (N)	Phenotype	Imputation Panel and Genome Build
APCDR-UGR (27)	Africa	Uganda	6407	DBP SBP	Africa genome panel, hg19
APCDR-DCC (27)	Africa	South Africa	1600	DBP SBP	Africa genome panel, hg19
APCDR-DDS (27)	Africa	South Africa	1165	DBP SBP	Africa genome panel, hg19
APCDR-AADM (27)	Africa	Nigeria Ghana Kenya	5231	DBP SBP	Africa genome panel, hg19
MVP–AFR	America	USA	59,933	DBP SBP	1000 Genome, hg19
UKB–AFR (28)	Europe	UK	6614	DBP SBP	1000 Genome, hg19

**Table 2:** Novel distinct variants identified using a meta-analysis approach.

Nearest Gene	Lead SNPs	Chr	BP	Effect Allele	Other Allele	Trait	Beta	SE	MAF	*p*-Value	Functional Consequence
*AC074290.1*	rs77534700	2	194657479	A	G	DBP	−0.0967	0.0176	0.0836	3.749x10^−8^	Intergenic variant
*MOBP*	rs562545	3	39536524	A	G	DBP	0.0593	0.0099	0.8973	1.823x10^−9^	Intron variant

**Table 3:** Novel variants of blood-pressure traits identified using multivariate methods.

Nearest Gene	Lead SNPs	Chr	BP	Effect Allele	Other Allele	HET_*p* Value	Functional Consequence
*DNAJC17P1/GLULP6* *GLULP6GLULP6* *GLULP6*	rs138493856	2	194678067	A	G	6.1322 × 10^−9^	Intergenic variant
*RRM2*	rs139235642	2	10278626	T	C	2.7981 × 10^−8^	Intron variant NMD transcript variant
*LOC105377644*	rs72619992	3	39407952	A	C	1.1339 × 10^−8^	Intron variant

## Data Availability

The data presented in this study are available on request from the corresponding author.

## Supplementary Materials

The following supporting information can be downloaded at: https://www.mdpi.com/1422-0067/24/3/2164/s1.
