# Correction: Udosen et al. Meta-Analysis and Multivariate GWAS Analyses in 80,950 Individuals of African Ancestry Identify Novel Variants Associated with Blood Pressure Traits. *Int. J. Mol. Sci.* 2023, *24*, 2164

**DOI:** 10.3390/ijms25074093

**Published:** 2024-04-07

**Authors:** Brenda Udosen, Opeyemi Soremekun, Abram Kamiza, Tafadzwa Machipisa, Cisse Cheickna, Olaposi Omotuyi, Mahmoud Soliman, Mamadou Wélé, Oyekanmi Nashiru, Tinashe Chikowore, Segun Fatumo

**Affiliations:** 1 The African Computational Genomics (TACG) Research Group, MRC/UVRI and LSHTM, Entebbe 7545, Uganda; brendaumoh6@gmail.com (B.U.); soremekun.opeyemi@mrcuganda.org (O.S.);; 2 The African Center of Excellence in Bioinformatics of Bamako (ACE-B), University of Sciences, Techniques and Technologies of Bamako, Bamako 3206, Mali; 3 H3Africa Bioinformatics Network (H3ABioNet) Node, Centre for Genomics Research and Innovation, NABDA/FMST, Abuja 901101, Nigeria; 4 Molecular Bio-Computation and Drug Design Laboratory, School of Health Sciences, Westville Campus, University of KwaZulu-Natal, Durban 4001, South Africa; soliman@ukzn.ac.za; 5 Malawi Epidemiology and Intervention Research Unit, Lilongwe P.O. Box 46, Malawi; 6 Hatter Institute for Cardiovascular Diseases Research in Africa (HICRA), Department of Medicine, University of Cape Town, Cape Town 7701, South Africa; 7 Population Health Research Institute, David Braley Cardiac, Vascular and Stroke Research Institute, Hamilton, ON L8L 2X2, Canada; 8 Department of Biological Sciences, Faculty of Sciences and Techniques, University of Sciences, Techniques and Technologies of Bamako, Bamako 3206, Mali; 9 Institute for Drug Research and Development, S.E. Bogoro Center, Afe Babalola University, Ado Ekiti 360101, Nigeria; 10 Sydney Brenner Institute for Molecular Bioscience, Faculty of Health Sciences, University of the Witwatersrand, Johannesburg 2050, South Africa; tinashe.chikowore1@wits.ac.za; 11 MRC/Wits Developmental Pathways for Health Research Unit, Department of Pediatrics, Faculty of Health Sciences, University of the Witwatersrand, Johannesburg 2050, South Africa; 12 Segun Fatumo, Department of Non-Communicable Disease Epidemiology, London School of Hygiene and Tropical Medicine, London WC1E 7HT, UK

In the original publication [1], the funding from United States’ Veteran Health Administration with grant numbers 5I01BX003360 and 1I01CX001897 awarded to Adriana M. Hung was not included. There was an error in the sample size stated in Figure 1 for the Million Veteran Program cohort. The sample size listed is 56,833 but the correct sample size is 59,933. The total sample size of the meta-analysis consequently increases to 80,950. Thus, all instances of the number 77,850 in the title, abstract, text, Table 1 and Figure 1 should be 80,950. The corrected Figure 1 and Table 1 appear below. The authors state that the scientific conclusions are unaffected. In the Acknowledgments, the authors would like to add an acknowledgment of the contributions of Jacklyn N. Hellwege, Ayush Giri, Adriana M. Hung and Todd L. Edwards of the Nashville VA and Vanderbilt University Medical Centers for leading the effort that generated the GWAS summary statistics of blood pressure in the Million Veteran Program included in this report. This correction was approved by the Academic Editor. The original publication has also been updated.

## Figures and Tables

**Figure 1:**
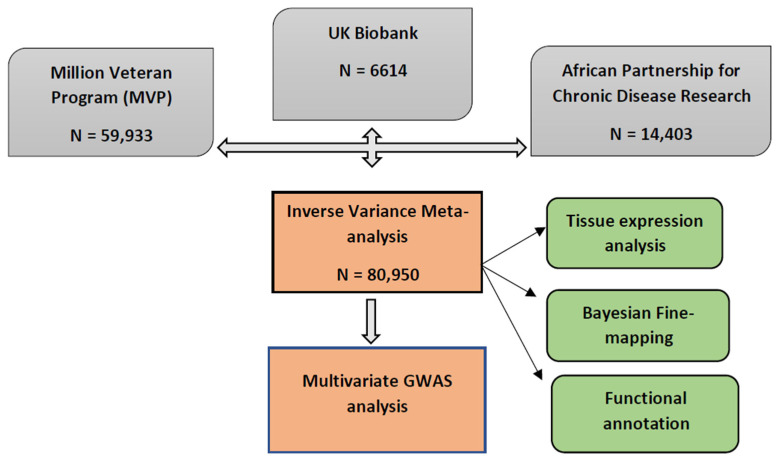
Study design schematic for discovery and validation of loci. APCDR; African Partnership for Control of Disease Research, UKB; United Kingdom Biobank, MVP; Million Veteran Program.

**Table 1:** Description of cohorts used in this study.

Cohort	Continent	Country	Sample Size (N)	Phenotype	Imputation Panel and Genome Build
APCDR-UGR (27)	Africa	Uganda	6407	SBP/DBP	African genome panel, hg19
APCDR-DCC (27)	Africa	South Africa	1600	SBP/DBP	African genome panel, hg19
APCDR-DDS (27)	Africa	South Africa	1165	SBP/DBP	African genome panel, hg19
APCDR-AADM (27)	Africa	Nigeria, Ghana, and Kenya	5231	SBP/DBP	African genome panel, hg19
MVP-AFR	N. America	USA	59,933	SBP/DBP	1k Genome, hg19
UKB-AFR (28)	Europe	UK	6614	SBP/DBP	1k Genome, hg19
