# Supplementary material for: Meta-Analysis and Multivariate GWAS Analyses in 80,950 Individuals of African Ancestry Identify Novel Variants Associated with Blood Pressure Traits
Source: Int J Mol Sci. 2023 Jan 21;24(3):2164. doi: 10.3390/ijms24032164 (PMC9916484; doi:10.3390/ijms24032164)

Supplementary 1

The supplementary materials have the following sections in order:

I.APCDR Study Cohort Descriptions

II. UK-BIOBANK Cohort Descriptions

III. MILLION VETERAN STUDY Cohort Descriptions

IV. Supplemental Figures

I. The UK Biobank Data

UKB is a large, population-based prospective study of ~500,000 richly phenotyped individuals aged 40-69 years recruited in 2006–2010, including BP measurements (Sudlow et al., 2015) established to allow detailed investigations of the genetic and non-genetic determinants of the diseases of middle and old age, with genotyping by customized Affymetrix Axiom Array chip array and imputation using a reference panel that merged the UK10K and 1000 Genomes Phase 3 panel as well as the Haplotype Reference Consortium (HRC) panel(Bycroft et al., 2017) yielding ~7 million SNPs (imputation quality score (INFO) ≥ 0.1 and MAF ≥ 1%). The UKB study has approval from the Northwest Multi-Centre Research Ethics Committee.

II African Partnership for Control of Disease Research Data

The APCDR is an international network of research groups that worked together to facilitate and promote collaborative research of chronic diseases across Africa. The GWAS was carried out using data from three cohorts under the APCDR. The Uganda Genome Resource (UGR) (A.G. et al., 2013); a total of 5,000 individuals were genotyped and 2,000 were sequenced from 9 ethnolinguistic groups. The cohort comprises all residents (52% aged >_13years, men and women both in equal proportions). This study was approved by Uganda Virus Research Institute Science and Ethics Committee and the Uganda National Council for Science and Technology (UNCST). The Durban Diabetes Study (DDS) is a population-based cross-sectional survey of an urban black population in the eThekwini Municipality (city of Durban) in South Africa. (Hird et al., 2013) The survey comprises of 1,165 individuals and was selected based on health, socioeconomic questionnaire data with standardized biophysical measurements, biomarkers for non-communicable and infectious diseases, lifestyle as well as genetic data. The DDS was approved

by the Biomedical Research Ethics Committee at the University of KwaZulu-Natal (reference: BF030/12) and the UK National Research Ethics Service (reference: 14/WM/1061). The Diabetes Case-Control study is a study of individuals with diabetes recruited from a tertiary hospital in Durban (n = 1,542). The Diabetes Case-Control (DCC) study was planned as a case-control study of type 2 diabetes to examine the epidemiology and genomics of type 2 diabetes and related cardiometabolic traits in a South African population. The collection started in 2009 and finished in 2013, however at the end of the study only cases (n = 1,600) had been recruited. The DCC was approved by the Biomedical Research Ethics Committee at the University of KwaZulu-Natal (reference: BF078/08) and the UK National Research Ethics Service (reference: 11/H0305/6). AADM is an ongoing genetic epidemiology study of type 2 diabetes and related traits in Africans which has been described in detail elsewhere (Rotimi et al., 2004). A total number of 5,231 individuals from the Africa America Diabetes Mellitus (AADM) study were included. Written informed consent was obtained from all participants. Demographic information was collected using standardized questionnaires across the AADM study centers in Nigeria (Ibadan, Lagos, and Enugu), Ghana (Accra and Kumasi), and Kenya (Eldoret). Anthropometric, medical history, and clinical examination parameters were obtained by trained study staff during a clinic visit. The population description summary can be found in table 3. Ethical approval was obtained from the Institutional Review Boards (IRB) of all participating institutions.

III Million Veteran Program (MVP)

MVP is an observational study cohort that aims to develop an infrastructure for future, broad-based research uses. Data were collected from participants using questionnaires, the VA electronic health record, and a blood sample for genomic and another testing. On August 3, 2015, followed by a steady state of ≈50 recruiting sites nationwide, N = 397,104 veterans have been enrolled. From the

total number of participants, N = 199,348 most participants (as expected) are male (92.0%) between the ages of 50 and 69 years (55.0). On the aspect of the self-reported race, white (77.2%) and African American (13.5%) populations are well represented. A total number of (n =56,833) participants were used for our genome-wide association studies of BP traits.

IV Supplementary Figure S1

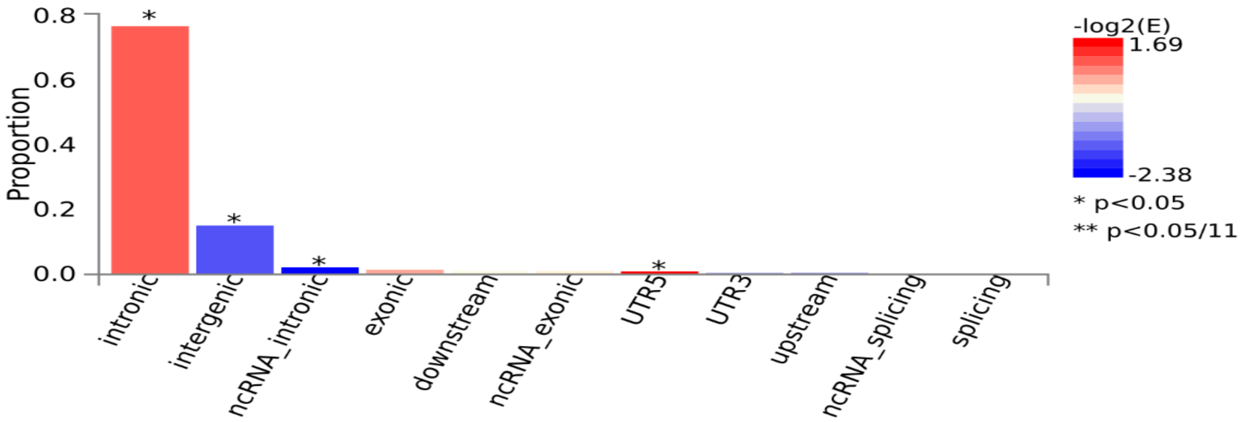

Supplementary Figure S1| Diagram showing the functional consequences of SNPs, majority of these SNPs is located in the intronic gene region followed by the Intergenic region.

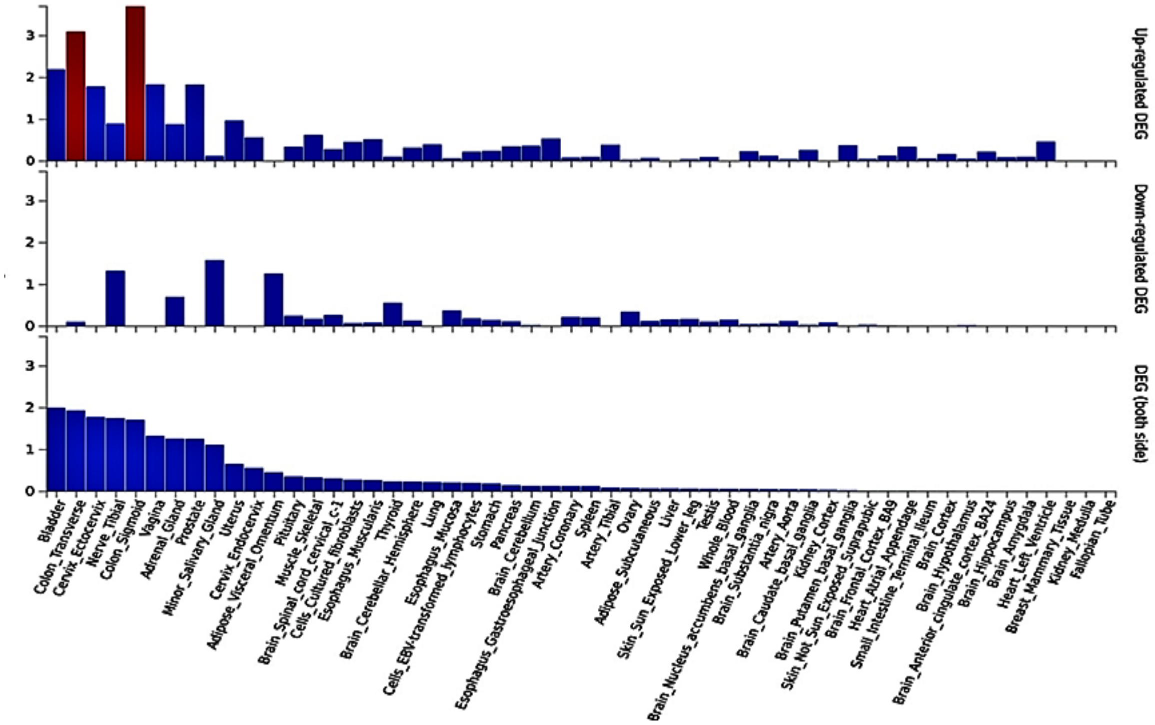

Supplementary Figure S2: GTEx v8 54 tissue types for SBP

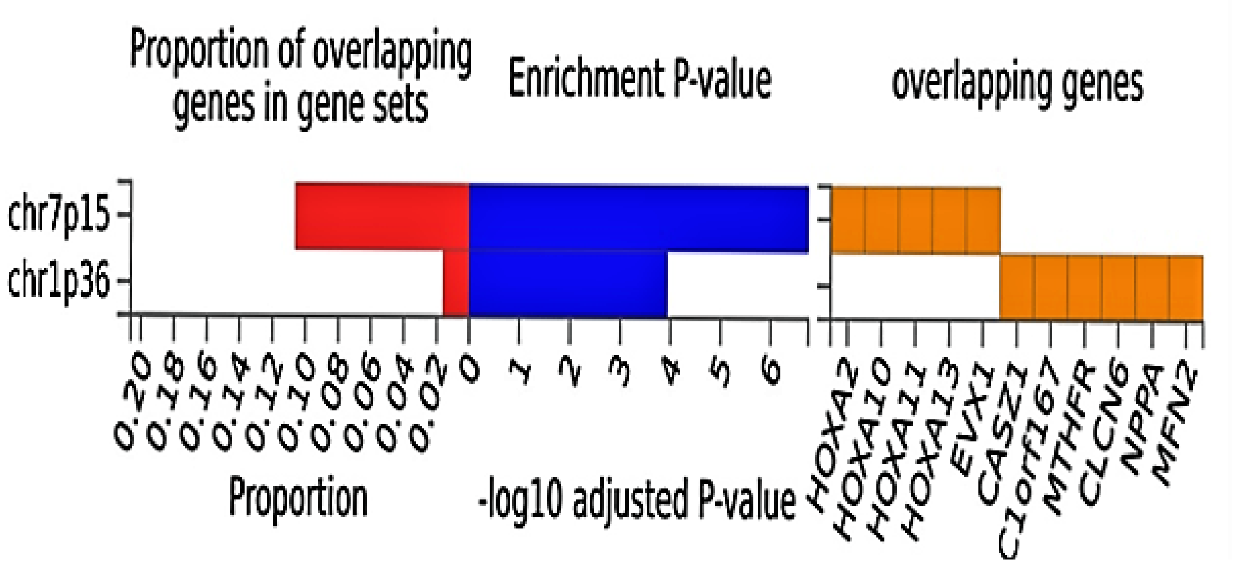

Supplementary Figure S3: Positional gene sets (MsigDB) for SBP

GeneSet	N	n	P-value	adjusted P	genes
REACTOME_CARDIAC_CONDUCTION	139	4	7.53e-6	1.13e-2	NPPA, ATP2B1, KCNK3, CACNA1D
REACTOME_MUSCLE_CONTRACTION	206	4	3.54e-5	2.65e-2	NPPA, ATP2B1, KCNK3, CACNA1D

Supplementary Figure S4:Reactome (MsigDB) for SBP

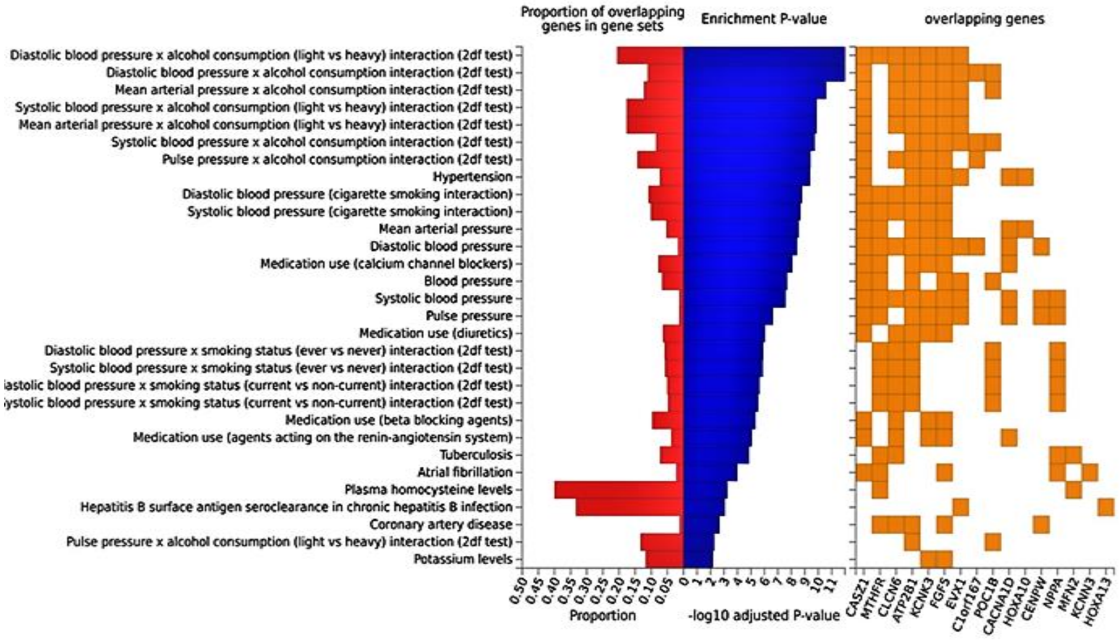

Supplementary Figure S5: GWAS catalog reported genes for SBP

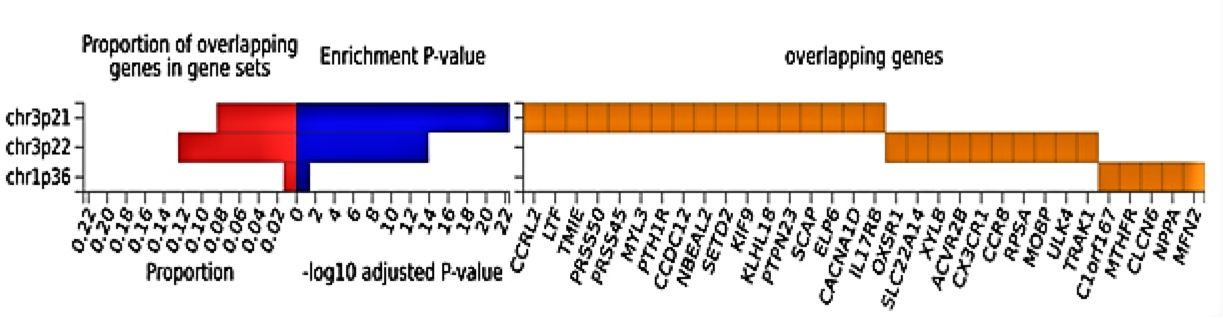

Supplementary Figure S6: Positional gene sets (MsigDB) for DBP

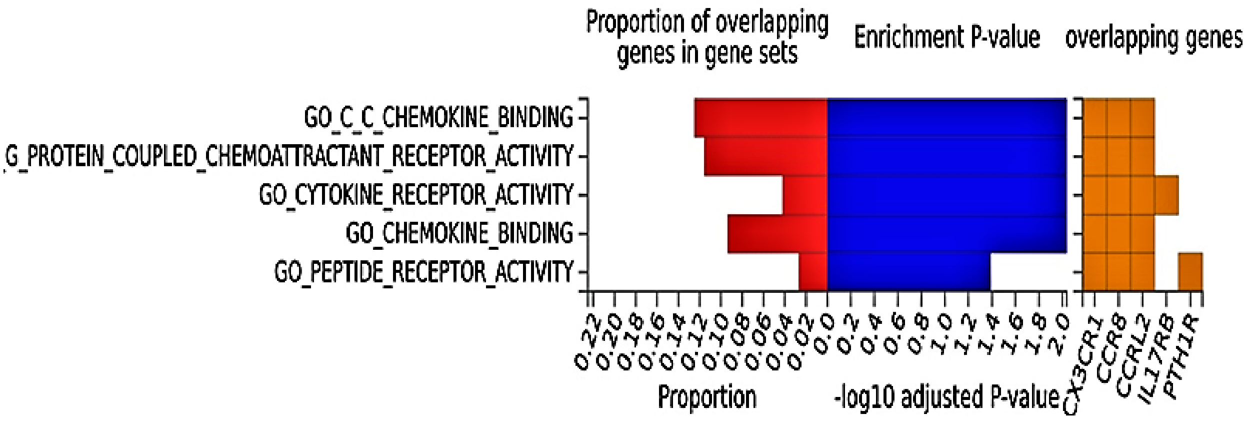

Supplementary Figure S7 : GO molecular functions (MsigDB c5) for DBP

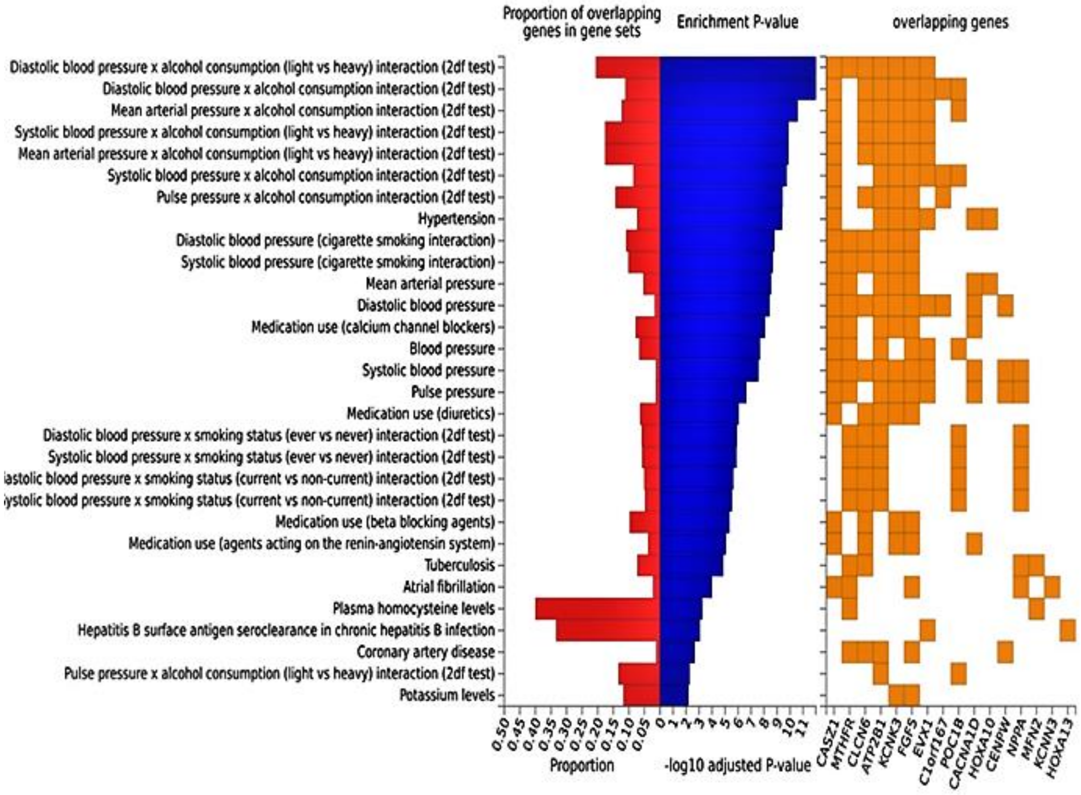

Supplement: Supplementary file 1 [file ijms-24-02164-s001.zip › ijms-2076201-supplementary.pdf]
